# Association between Daily Niacin Intake and Glaucoma: National Health and Nutrition Examination Survey

**DOI:** 10.3390/nu13124263

**Published:** 2021-11-26

**Authors:** Teerajet Taechameekietichai, Sunee Chansangpetch, Pimnara Peerawaranun, Shan C. Lin

**Affiliations:** 1 University Hospital of North Tees, North Tees and Hartlepool NHS Foundation Trust, Stockton-on-Tees, Durham TS19 8PE, UK; christerajet@live.com; 2 Glaucoma Research Unit, Faculty of Medicine, Chulalongkorn University and King Chulalongkorn Memorial Hospital, Thai Red Cross Society, Bangkok 10330, Thailand; 3 Mahidol Oxford Tropical Medicine Research Unit, Faculty of Tropical Medicine, Mahidol University, Bangkok 10400, Thailand; pimnara@tropmedres.ac; 4 Glaucoma Center of San Francisco, San Francisco, CA 94105, USA; sl@glaucomasf.com

**Keywords:** niacin, glaucoma, nicotinamide, NHANES

## Abstract

Background and Aims: To determine the relationship between dietary intake of niacin and glaucoma using the data from the 2005 to 2008 National Health and Nutrition Examination Survey (NHANES). Methods: Subjects aged 40 years and older who participated in the dietary intake interview and vision health questionnaire of NHANES were included in the study. Glaucoma diagnosis by self-report was utilized. Additionally, glaucoma diagnosis by fundus imaging and International Society Geographical and Epidemiological Ophthalmology (ISGEO) criteria was used in subjects with available qualified retinal imaging. Survey logistic regression analyses were used to examine the association between daily niacin consumption and glaucoma. Results: A total of 5768 participants were included in the study. There was a significant decrease in the crude odds of self-reported glaucoma in the third (OR 0.57, 95% Cl 0.43–0.76; *p* < 0.001) and fourth (OR 0.57, 95% Cl 0.37–0.90; *p* = 0.018) quartiles of daily niacin consumption, which equated to 21.01 to 28.22 mg/day and greater than 28.22 mg/day, respectively. A similar trend was found using fundus imaging of subjects with niacin intake in the third (OR 0.42, 95% Cl 0.25–0.72; *p* = 0.002) and fourth (OR 0.36, 95% Cl 0.20–0.67; *p* = 0.002) quartiles. After adjusting for covariates, the odds of glaucoma based on fundus imaging remained significantly lower for niacin intake in the third (OR 0.49, 95% Cl 0.28–0.87; *p* = 0.016) and fourth (OR 0.48, 95% Cl 0.26–0.89; *p* = 0.022) quartile levels. Using ISGEO criteria, there was no significant association between glaucoma and daily niacin consumption. Conclusions: Greater niacin intake may be associated with a lower chance of developing glaucoma.

## 1. Introduction

Glaucoma is a condition that involves loss of retinal ganglion cells (RGC) and axons, resulting in chronic and progressive optic neuropathy. The features of glaucoma include characteristic optic disc changes and visual field defects [1]. Globally, glaucoma is one of the leading causes of irreversible blindness, with the estimate of more than 70 million affected individuals [2]. While the pathophysiology of glaucoma is still not fully understood, an increase in the intraocular pressure (IOP) is one of the main risk factors contributing to the development of glaucoma [3,4]. Nevertheless, many patients with glaucoma continue to have disease progression despite being on IOP-lowering treatments or having relatively low IOP, prompting the search to find other possible means, such as neuroprotection, to slow or cease the progression of glaucomatous damage [5,6,7,8].

The optic nerve is anatomically an extension of the central nervous system (CNS), and consists of the RGC axons. Up to ninety percent of the RGC extend to the lateral geniculate nucleus, which then transmits information to the visual cortex [9]. Recent research suggests an association between glaucoma and neurodegeneration found in the CNS of primates and humans [10,11,12]. These glaucoma-associated neurodegeneration processes share a lot of similarities with other CNS conditions, such as amyotrophic lateral sclerosis and Alzheimer’s disease [13,14,15].

There is also growing evidence that supports a link between vitamin intake and the function of the nerve cells [16,17]. Currently, there is accumulating evidence demonstrating the importance of niacin, also known as vitamin B3, in the development and maintenance of CNS structures [18,19]. Furthermore, mitochondrial dysfunction and metabolite depletion, especially the reduction in total nicotinamide adenine dinucleotide (NAD), have been found in the early stages of glaucoma [20]. Therefore, it was proposed that restoring NAD, with its precursor niacin, could provide a neuroprotective effect against glaucoma [21].

This paper aims to determine the relationship between dietary intake of niacin and glaucoma using the National Health and Nutrition Examination Survey (NHANES), a large population-based study in the United States (US). Identifying other modifiable risk factors will be beneficial in providing possible additional treatment to patients suffering from glaucoma.

## 2. Materials and Methods

### 2.1. Sample and Population

We examined publicly available data from the 2005 to 2008 NHANES, an annual cross-sectional series of interviews and physical examinations conducted on approximately 5000 members of the civilian, non-institutionalized US population each year [22]. The program is administered by the National Center for Health Statistics (NCHS), which is part of the US Centers for Disease Control and Prevention. NHANES uses a stratified multistage sampling design with a weighting scheme in order to accurately estimate disease prevalence in the US population. The period from 2005 to 2008 was selected, as surveys carried out during that time included questionnaires and data relating to the presence or absence of glaucoma.

The primary outcome for this study was the presence of self-reported glaucoma. Two additional glaucoma outcomes—glaucomatous optic neuropathy based on retinal imaging, and glaucoma diagnosis using the International Society for Geographical and Epidemiological Ophthalmology (ISGEO) criteria—were also analyzed.

**Figure 1:**
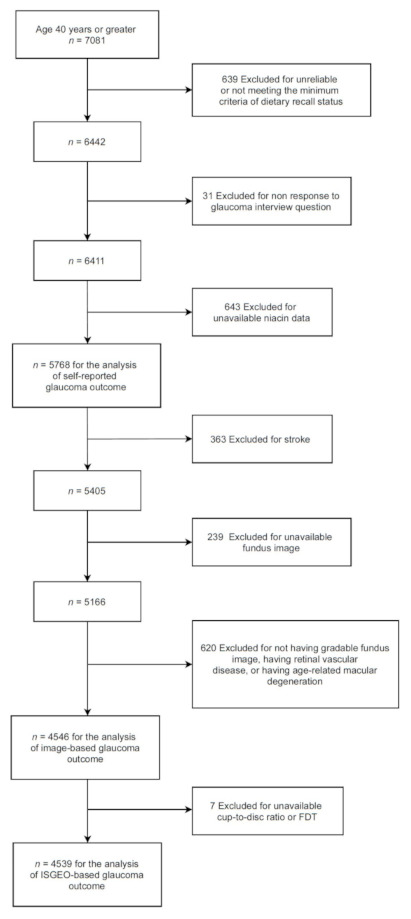
Flow diagram illustrating selection of study participants. ISGEO: International Society of Geographical and Epidemiologic Ophthalmology.

We included 7081 subjects who were 40 years or older and underwent the dietary interview. Among eligible participants, 639 and 643 subjects were excluded for incomplete data in at least one of two dietary interviews, and for unavailable niacin data, respectively. After exclusion, we included the eligible subjects who also participated in the vision questionnaire for the analysis of self-reported glaucoma outcome, who had retinal imaging examination for the analysis of image-based glaucoma outcome, and who had retinal imaging and frequency doubling technology (FDT) testing for the analysis of ISGEO-based glaucoma outcome (Figure 1).

### 2.2. Measures

The predictors were daily niacin intakes, which were available in the “Dietary Interview-Total Nutrient Intakes dataset”. The nutrient data was obtained from the dietary interview component, which is called What We Eat in America (WWEIA). This survey was conducted as a partnership between the US Department of Agriculture (USDA) and the US Department of Health and Human Services (DHHS). Under this partnership, DHHS’ NCHS is responsible for the sample design and data collection, and USDA’s Food Surveys Research Group is responsible for the dietary data collection methodology, maintenance of the databases used to code and process the data, and data review and processing [23]. In brief, all eligible NHANES examinees receive two 24-h dietary recall interviews regarding the types and amounts of foods consumed during the 24-h period prior to the interview (midnight to midnight). The first dietary recall interview is conducted in-person in the Mobile Examination Center, and the second interview is carried out by telephone 3 to 10 days later.

The total nutrient intakes dataset provided a summary record of total nutrient intakes for each individual. Each total intake record includes the number of days of complete intake, day of the week of the intake, and daily aggregates of food energy. The nutrients/food components from all foods are calculated using USDA’s Food and Nutrient Database for Dietary Studies (FNDDS). The underlying nutrient values for FNDDS were based on values in the USDA National Nutrient Database for Standard Reference, produced by USDA’s Nutrient Data Lab [24]. FNDDS values are updated for every 2-year WWEIA/NHANES release cycle. FNDDS 4.1 corresponds with WWEIA 2007–2008. FNDDS 3.0 corresponds with WWEIA 2005–2006.

### 2.3. Outcome Variables

The prevalence of self-reported glaucoma was assessed using data collected from the following question in the vision questionnaire, sent out to individuals aged 40 years or older: “Have you ever been told by an eye doctor that you have glaucoma, sometimes called high pressure in your eyes?”. Respondents who answered “Don’t know” and those who gave no answer were excluded. A total of 5768 subjects remained for the purpose of analysis of this outcome variable.

Retinal imaging and FDT visual field testing were offered to subjects aged 40 years or older, unless they were unable to see light with both eyes open, or had an eye infection. For retinal imaging, two 45° non-mydriatic digital images were obtained from both eyes using the Canon Non-Mydriatic Retinal Camera CR6-45NM (Canon, Tokyo, Japan). The first image was centered on the macula, and the second was centered on the optic nerve. The images were initially read at the University of Wisconsin with an emphasis on features relevant to diabetic retinopathy and age-related macular degeneration. In 2012, the retinal images of subjects with a cup-to-disc ratio (CDR) greater than or equal to 0.6 were re-read by ophthalmologists based at Johns Hopkins University with attention to features relevant to glaucoma. Glaucoma in each eye was classified as “no, possible, probable, definite, or unable to assess.” If at least two of three graders provided the same grade, and the third grader was within one level, then that grade was assigned to the image. If at least two of the graders did not agree, or if the third grader was off by two or more levels, then the image was re-read in the presence of all three graders in order to achieve consensus [25]. In this analysis, participants were subsequently categorized as having glaucoma if they had probable or definite glaucoma in either eye. Those who could not successfully complete retinal imaging in both eyes were excluded, and 4546 remained in the analysis of this outcome variable.

The Humphrey Matrix Visual Field Instrument was utilized for FDT perimetry, and the N-30-5 FDT screening protocol was used in the assessment. The FDT tests were performed in a dark room by trained investigators. During the assessment, nineteen visual field locations of each eye were tested. Individual locations were tested until a response was received. Each of the participant’s eyes were tested twice. Moreover, a practice test was run prior to the actual test to determine whether the participant understood the test procedures. Identification of an abnormal visual field occurred when both the first and second test showed that at least two locations fell below a 1% threshold level, and at least one failed location was the same in both tests (2-2-1 algorithm). To check for the reliability of each test, three false-positive and blind-spot checks were carried out at random. The FDT result for each participant was subsequently categorized as normal, positive, insufficient, or unreliable. Participants with insufficient or unreliable FDT were then removed from the study [26].

The ISGEO criteria, which are consistent with the Rotterdam criteria, utilized both optic nerve appearance and glaucomatous visual field defect (GVFD) information [27,28,29]. The CDR data from the retinal imaging subsection and visual field data from the FDT subsection were assessed, and ISGEO diagnosis categories 1 and 2 were utilized for classification in the study. A positive GVFD was indicated when two of the N30-5 FDT tests detected two or more abnormal points, where one must be consistent in two consecutive tests, in the same eye [30,31].

In brief, the diagnosis of glaucoma was made if any of the following were present:

(1) CDR > 99.5th percentile for the mean NHANES population in either eye, regardless of the FDT results.
(2) CDR asymmetry between eyes > 99.5th percentile for the mean NHANES population, regardless of the FDT results.
(3) CDR > 97.5th percentile for the mean NHANES population in either eye with positive FDT result in the same eye.
(4) CDR asymmetry between eyes > 97.5th percentile for the mean NHANES population with positive FDT result in at least one eye.

Subjects with stroke, retinal vascular diseases, or age-related macular degeneration, which can affect glaucoma interpretation from fundus imaging and FDT, were excluded from the analysis. A flow diagram illustrating a section of study participants is shown in Figure 1.

### 2.4. Data Analysis

Descriptive statistics were calculated with means and standard deviations for continuous variables and proportions for categorical variables. The distribution of possible confounding variables between subjects, with and without glaucoma based on self-reported criteria, were compared using design-adjusted Rao-Scott Pearson-type chi-squared tests, and Wald tests for categorical and continuous variables, respectively. The standard errors of population estimates were calculated using Taylor linearization methods. The usual intake of niacin was categorized into quartiles, and were then treated as predictors for the presence of glaucoma by each set of criteria. The model adjusted for age, gender, ethnicity, education level, and diabetes. All analyses were weighted for the complex survey design. All tests were two-sided, and *p* < 0.05 was considered statistically significant. This part of the analyses was performed using Stata 16.1 (StataCorp LP, College Station, TX, USA).

## 3. Results

The NHANES data, from 2005 to 2008, yielded a total of 5768 subjects aged 40 years or older who had an available glaucoma interview response and reliable niacin data. Out of these subjects, the number of self-reported glaucoma was 393, which represented 5.3% of the 240,790,568 weighted population (Supplement Table S1). The number of subjects with glaucoma assessed by fundus imaging and ISGEO criteria is 125 (1.8% in the weighted population) and 111 (1.6% in the weighted population), respectively (Supplement Table S1). As displayed in Table 1, the mean age of subjects with self-reported glaucoma is greater than those without glaucoma (*p* < 0.001). Compared to the subjects without glaucoma, a higher proportion of subjects with diabetes (*p* < 0.001) and non-Hispanic black race (*p* = 0.02) are found in the self-reported glaucoma group. Furthermore, a proportion of subjects with daily total energy intake (*p* < 0.001) and more than a high school educational level (*p* = 0.01) were significantly higher in the self-reported no glaucoma group.

The mean daily niacin consumption in all eligible subjects was 24.18 (SE 0.27) mg/day. The mean daily niacin consumption in the group with self-reported glaucoma (21.14 [SE 0.64] mg/day) was significantly lower than in subjects without glaucoma (24.35 [SE 0.27] mg/day) (*p* < 0.001). Moreover, we divided the daily niacin consumption into first (<15.33 mg/day), second (≥15.33 to <21.01 mg/day), third (≥21.01 to <28.23 mg/day), and fourth (≥28.23 to 161.05 mg/day) quartiles for additional analysis.

We examined the potential relationship between daily niacin intake and the prevalence of self-reported glaucoma. There was a significant decrease in the odds of glaucoma in the third (OR 0.57, 95% Cl 0.43–0.76; *p* < 0.001) and fourth (OR 0.57, 95% Cl 0.37–0.90; *p* = 0.018) quartiles of daily niacin consumption as compared to the first quartile. Additionally, the unadjusted odds of self-reported glaucoma showed a decreasing trend with higher quartiles of niacin intake (*p* trend = 0.001). After adjusting for covariates (model 1: adjusted for age and gender; model 2: adjusted for age, gender, total energy intake, race, educational level, diabetes), subjects with niacin intake in the third quartile were less likely to self-report glaucoma (model 1: OR 0.63, 95% Cl 0.46–0.87; *p* = 0.006; model 2: OR 0.95, 95% CI 0.61–1.50; *p* = 0.833) when compared to the first quartile.

We further investigated the association of daily niacin intake and the prevalence of glaucoma identified by fundus images (Supplement Table S2). We found that subjects with niacin intake in higher quartiles were significantly associated with lower odds of glaucoma in an unadjusted model (*p* trend< 0.001). Table 2 illustrates that the crude odds of glaucoma disease is the least in the fourth quartile (OR 0.36, 95 %Cl 0.20–0.67; *p* = 0.002). Similarly, in adjusted models, we identified that subjects with niacin intake in the fourth quartile are the least likely to have glaucoma identified by fundus imaging. We also performed trend analysis and found that the adjusted odds of glaucoma were reduced with greater daily niacin consumption in both models (model 1: *p* trend < 0.001, model 2: *p* trend = 0.040).

Using ISGEO criteria to identify glaucoma, we utilized the same analysis method as previously mentioned (Supplement Table S3 and Supplement Table S4). In both the unadjusted and adjusted models, no statistically significant association between daily niacin consumption and glaucoma was found (Table 2).

## 4. Discussion

Using a US national population-based sample of adults 40 years and older, our study identified an association between daily niacin consumption and glaucoma determined by a self-reported method and fundus imaging. Participants with a niacin intake greater than 21.00 mg/day (quartiles 3 and 4) had a significantly lower risk of glaucoma compared to those with niacin intake of less than 15.33 mg/day (quartile 1).

Niacin, a form of vitamin B3, includes two vitamers (nicotinamide (NAM) and nicotinic acid (NA)) [32] which are involved in the synthesis pathway of NAD [19]. It is worth noting that vitamin B3 can refer to either NA, NAM, or nicotinamide riboside, and that the bacteria in the gut can turn both nicotinamide riboside and NAM into NA [33].

The role of NAD in glaucoma has been studied in several laboratory investigations. The first possible mechanism for protection is related to the mitochondrial energy production pathway. Many studies have demonstrated a possible association of mitochondrial dysfunction with glaucoma [34,35,36]. As RGCs are responsible for transducing visual information from the retina to the brain, they require a relatively large amount of energy. Thus, reduction of NAD, via NAD-consuming enzymes, may limit adenosine triphosphate (ATP) production, and result in the failure to provide sufficient energy for sustaining the health of the cell, ultimately leading to the degeneration of RGCs [37].

Another potential benefit of NAD to glaucoma is the prevention of axonal degeneration. Studies looking at neurodegeneration in glaucoma using an animal model suggest that Wallerian degeneration is a possible mechanism for distal degeneration [38,39]. Evidence in mice with a Wallerian degeneration slow (Wlds) allele have suggested that there is protection against axonal degeneration via an NAD-dependent mechanism [40]. Araki et al. found that variation in the nicotinamide mononucleotide adenylyltransferase-1 (NMNAT1) portion of the fusion protein is responsible for the phenotype of Wlds [41]. 

Due to these possible mechanisms, restoring the NAD precursors, such as NAM, could help to enhance the resistance of RGC against glaucomatous neurodegeneration [20,21]. Supporting this hypothesis, studies have also shown that NAM treatment helped to improve NAD level, and provide a neuroprotective effect against glaucoma [20,21,42]. An administration of a NAM dose at 550 mg/kg/day, equivalent to approximately 2.7 g/day for a 60 kg human, in DBA/2J mice was shown to increase NAD level, and provide a robust neuroprotective effect [21]. In comparison to a NAM dose of 550 mg/kg/day, a higher dose of 2000 mg/kg/day (comparable to 9.8 g/day for a 60 kg human) demonstrated a greater neuroprotective effect, where no optic nerve damage was found in 93% of eyes [43]. Similarly, in an in vivo model using OCT imaging in rats pre-treated with NAM, it was shown that the lowest dose for neuroprotection is 200 mg/kg/day, which converts to 1.9 g/day in a 60 kg human [42]. Rats pre-treated at a higher dose (400 and 800 mg/kg/day for an equivalent of 4 and 8 g/day in a 60 kg human, respectively) appear to have significantly decreased RCG loss and nuclear shrinkage in a dose-dependent pattern [42]. These studies indicate a possible relationship between the dose of NAM and the neuroprotective effect in glaucoma.

Regarding clinical evidence on NAM supplementation, a randomized control trial by Hui et al. demonstrated that NAM use for 12 weeks could improve inner retinal function in glaucoma, measured by electroretinography and perimetry [44]. The NAM doses used in the study were 1.5 g/day for 6 weeks, followed by 3.0 g/day for another 6 weeks [44]. The high doses in their study were obtained from the preclinical evidence and clinical trials for other indications. Their results support the potential mechanism of NAM in the neuroprotection of RGCs. Table 3 summarizes the preclinical and clinical experimental studies of niacin and glaucoma.

Though supplementing with NAD or its precursors can possibly improve RGC function, inadequate intake of niacin may cause axons to become more vulnerable, making them more susceptible to glaucomatous damage. Nzoughet et al. investigated the plasma concentration of NAM in individuals with primary open-angle glaucoma (POAG), and discovered that individuals with POAG have a lower NAM level than the controls [37]. As past studies have indicated that the plasma level of NAM is dependent on the oral dose [45,46], our results indirectly agree with Nzoughet et al.’s findings that lower daily intake of niacin is associated with an increased risk of glaucoma. However, a recent review highlighted that there is no strong evidence of niacin in reducing the risk of glaucoma in the human study; thus, the relation between serum vitamin B levels and glaucoma in humans, and the relation between clinical effects, administered dose, and the serum levels in different types of glaucoma should be further investigated [47].

Our results show that the odds of having glaucoma were nearly two times lower in subjects with a daily intake of niacin in the third and fourth quartiles, as compared to the first and second quartiles. Despite not being statistically significant, subjects who had niacin intake in the fourth quartile also had odds of glaucoma diagnosed by ISGEO approximately half of those in the second quartile. The current recommended dietary allowance (RDA) of niacin equivalent is 16 mg/day for men and 14 mg/day for women [48]. Our results have shown that the mean daily niacin intake of individuals aged 40 years or older was 24.18 mg/day, which is greater than the RDA dosage. Though possible adverse effects of niacin include flushing, chills, and gastrointestinal side effects [49], several studies have demonstrated that an intake of nearly 1 g (or up to 3 g) of NAM daily is well tolerated even in long-term administration [50]. Nevertheless, individuals with daily niacin intake within the first quartile (<15.33 mg/day) are more likely to have an intake less than the RDA, which may help to explain why they are more likely to have glaucoma. These findings indicate an association between daily intake of niacin and glaucoma, possibly due to increasing RGC susceptibility to degeneration or damage from IOP.

A randomized trial had shown that oral nicotinamide riboside, another form of niacin, could increase whole blood NAD in a dose-dependent manner [51]. Moreover, Zhang et al. have also demonstrated that prophylactic systemic treatment with nicotinamide riboside could help to increase retinal NAD levels, and provide a neuroprotective effect to the RGCs in the acute and chronic mouse glaucoma model [52]. As nicotinamide riboside has better bioavailability than NAM in humans [53], and is safe and well-tolerated [54,55,56]. Further study is needed to explore whether nicotinamide riboside could be a potential alternative in glaucoma treatment.

**Table 3:** Summary of preclinical and clinical experimental studies.

Study	Design	Subjects (*n*)	Niacin Form	Human Doses or Equivalent	Outcomes	Results
Williams et al. [21]	Animal study	DBA/2J mice	NAM doses at 550 and 2000 mg/kg/day	2.7 g/day and 9.8 g/day in a 60 kg human	Optic nerve degeneration assessed by paraphenylenediamine staining	Dietary supplementation with NAM reduces vulnerability to glaucoma.
Tribble et al. [42]	Animal study	C57BL/6J mice	NAM dose at 200, 400 and 800 mg/kg/day	1.9, 4, and 8 g/day in a 60 kg human	Loss of neuroretinal rim by OCT RGC loss and nuclear shrinkage assessed by RBPMS and DAPI labelling of flat mounts	NAM buffers against metabolic and bioenergetic insufficiency, and provides neuroprotection against glaucoma-related stresses.
Zhang et al. [52]	Animal study	C57BL/6J mice for optic nerve crush (acute model) and DBA/2J mice intracameral microbead injection (chronic model) experiments	Intraperitoneal injection of NR at 1000 mg/kg	N/A	RGC survival, assessed by counting cells in retinal flatmounts immunostained for Brn3a+ RGC function was assessed by pERG Suppressed retinal inflammation assessed by immunofluorescence staining of retinal fixed sections for (GFAP)	Prophylactic systemic treatment with NR is protective in acute and chronic mouse models of RGC damage.
Hui et al. [44]	Crossover randomized clinical trial	Early to moderate treated glaucoma (*n* = 57)	NAM doses at 1.5 g/day for 6 weeks, followed by 3.0 g/day for another 6 weeks		Inner retinal function measured by electroretinography photopic negative response parameters	Participants received NAM have a better improvement in inner retinal function relative to control.

NAM, nicotinamide; NAD+, nicotinamide adenine dinucleotide; pERG, pattern electroretinograms; GFAP, glial fibrillary acidic protein; RBPMS, RNA-binding protein with multiple splicing; NR, nicotinamide riboside; RGC, retinal ganglion cell; N/A, not available.

Our results are consistent with Jung et al.’s study in the Korean population showing that subjects with glaucoma have a significantly lower dietary intake of niacin [57]. Jung et al. analyzed the Korean National Health and Nutrition Examination Survey V (2008–2012), and divided all nutrient intake into quartiles: the quartiles 1, 2, 3, and 4 for niacin were <10.15, ≥10.15, ≥14.42, and ≥20.49 mg/day, respectively. In the study, the odds of glaucoma are the lowest in quartile 4 (OR: 0.60). Interestingly, the IOPs of all four quartiles were similar to each other, at about 14 mmHg. The study further looked at subjects with IOP equal to or less than 21 mmHg, and found that the highest quartile (Q4) had the lowest odds ratio for glaucoma, at 0.63. This finding indicated that individuals with higher daily niacin intake have lower odds of glaucoma, independent of IOP, which suggests that niacin’s protective effect against glaucoma is not via IOP reduction. Compared to the study by Jung et al., our study used a different population sample, had a greater number of outcomes, and used nutritional data that was based on dietary interviews conducted twice. To the best of our knowledge, no other studies have looked at the relationship between niacin and glaucoma in a different population, and this is the first study to analyze the association using the NHANES data.

The primary outcome of our study was self-reported glaucoma. However, there are a few potential limitations related to this outcome. First, the subjects with self-reported glaucoma were not confirmed by ophthalmologic examination, which may result in misclassification bias. Additionally, subjects may have a lack of awareness regarding their ophthalmic conditions because of lack of past diagnoses, memory problems, or poor eye health literacy. It is worth noting that studies have demonstrated that self-reported glaucoma has a low sensitivity, but high specificity for the condition [58]. As the IOP of the participants is not available from the NHANES data, another potential limitation for this study is that we could not include the IOP in the diagnosis of glaucoma. Therefore, our study included two additional outcomes—fundus imaging and ISGEO criteria—where the ISGEO criteria are less likely to be subjected to recall bias or misclassification bias. Though the results from fundus imaging were similar to the self-reported ones, the analyses using the ISGEO criteria were all statistically non-significant. The negative findings could be due to a smaller number of participants that had both gradable fundus imaging and qualified FDT results. In addition, there is a relatively small number of glaucoma cases diagnosed by these criteria, which could lead to inadequate statistical power. This could be from the fact that structural changes, which can be detected from fundus imaging, often precede glaucomatous visual field changes [59]. The diagnosis by the ISGEO criteria require the presence of abnormalities of both structure and function, and thus, was more stringent. It is possible that mild glaucoma cases were not included by this criterion.

One of the strengths of this study is that the subjects used were representative of patients with glaucoma in the general US population. However, our results could have been confounded by unmeasured factors, such as lifestyle, that associate with dietary intake. This study also contains other limitations due to its observational nature. First, the cause-and-effect relationship between niacin intake and glaucoma cannot be established from our study. Therefore, further controlled trials or epidemiological cohort studies would be required to confirm the benefit of niacin in glaucoma. The dietary intake of the participants was based on a food frequency questionnaire, which is subject to recall bias and misclassification bias—even though this should not affect glaucoma and non-glaucoma groups differently. The bioavailability of niacin in each participant is also different, which may lead to inaccuracy in assessing the dietary effects. Hence, to investigate the direct relationship between niacin and glaucoma, a serum analysis may be required in future studies. Furthermore, a comprehensive ophthalmic examination to distinguish between different types of glaucoma was not conducted in NHANES, and the link between niacin intake and different types of glaucoma may vary. We feel that to gain a better understanding of the potential role of niacin in glaucoma treatment, it would be important for further studies to investigate the types of glaucoma for which niacin would be most beneficial.

## 5. Conclusions

In conclusion, we found that an increased level of daily niacin intake was associated with a lower likelihood of glaucoma, by self-report and fundus imaging, in the US population. Our study also suggests that a certain threshold of niacin dosage is required for the potential effect of glaucoma prevention to be the greatest. Further research is needed to determine the precise dose and time relationship in which the possible beneficial effect on the optic nerve is optimal.

## Figures and Tables

**Table 1:** Comparison of demographics and characteristics of participants with or without self-reported glaucoma.

Characteristics	Self-Reported Glaucoma	Self-Reported No Glaucoma	*p* Value ^a^
	Estimated Mean/% Proportion (SE)	Estimated Mean/% Proportion (SE)	
Age, years	65.8 (1.17)	56.9 (0.38)	<0.001 ^b^
Gender			
Male	49.2 (0.04)	45.4 (0.01)	0.32
Female	50.8 (0.04)	54.6 (0.01)	
Race/ethnicity			
Non-Hispanic White	72.6 (0.04)	78.1 (0.02)	0.02
Non-Hispanic Black	16.4 (0.03)	9.8 (0.01)	
Mexican and Hispanic	6.2 (0.01)	8.0 (0.01)	
Other races	4.8 (0.02)	4.1 (0.01)	
Educational level			
Less than high school	24.4 (0.03)	17.3 (0.01)	0.01
High school graduation or equivalent	30.8 (0.04)	26.6 (0.01)	
More than high school	44.8 (0.04)	56.1 (0.02)	
Annual household income, USD ($)			
<20,000	18.0 (0.05)	14.0 (0.01)	0.32
≥20,000	82.0 (0.05)	86.0 (0.01)	
Diabetes			
No	76.2 (0.02)	89.2 (0.01)	<0.001
Yes	23.8 (0.02)	10.8 (0.01)	
Daily total energy, kcal	1690.7 (46.3)	2015.1 (19.6)	<0.001 ^b^

SE = standard error. ^a^ Design-adjusted Rao-Scott Chi-squared test, ^b^ Adjusted Wald test.

**Table 2:** Associations between daily niacin intake and glaucoma diagnosis by self-report, fundus imaging, and ISGEO criteria.

	Daily Niacin Intake	Glaucoma	Participants	Crude OR (95% CI)	*p* Value	Model 1 OR (95% CI)	*p* Value	Model 2 OR (95% CI)	*p* Value
Self-reported	Quartile 1	122	1442	Ref		Ref		Ref	
	Quartile 2	111	1442	1.08 (0.71 to 1.65)	0.704	1.15 (0.75 to 1.76)	0.518	1.42 (0.89 to 2.27)	0.133
	Quartile 3	83	1442	0.57 (0.43 to 0.76)	<0.001	0.63 (0.46 to 0.87)	0.006	0.95 (0.61 to 1.50)	0.833
	Quartile 4	77	1442	0.57 (0.37 to 0.90)	0.018	0.73 (0.44 to 1.20)	0.207	1.39 (0.74 to 2.58)	0.292
	*p trend*				0.001		0.054		0.557
Fundus Image	Quartile 1	35	1036	Ref		Ref		Ref	
	Quartile 2	34	1091	0.78 (0.41 to 1.47)	0.424	0.80 (0.43 to 1.49)	0.470	0.77 (0.40 to 1.48)	0.415
	Quartile 3	29	1188	0.42 (0.25 to 0.72)	0.002	0.43 (0.25 to 0.75)	0.004	0.76 (0.21 to 0.99)	0.050
	Quartile 4	27	1231	0.36 (0.20 to 0.67)	0.002	0.42 (0.23 to 0.75)	0.005	0.50 (0.23 to 1.05)	0.067
	*p trend*				<0.001		<0.001		0.040
ISGEO criteria	Quartile 1	29	1034	Ref		Ref		Ref	
	Quartile 2	33	1089	1.18 (0.50 to 2.80)	0.691	1.20 (0.51 to 2.79)	0.669	1.20 (0.56 to 2.58)	0.624
	Quartile 3	26	1187	0.60 (0.27 to 1.32)	0.197	0.59 (0.26 to 1.33)	0.196	0.63 (0.34 to 1.17)	0.140
	Quartile 4	23	1229	0.60 (0.24 to 1.49)	0.258	0.62 (0.24 to 1.63)	0.321	0.72 (0.34 to 1.54)	0.387
	*p trend*				0.105		0.159		0.144

Model 1 adjusted for age and gender. Model 2 adjusted for age, gender, race, total energy intake, educational level, diabetes. ISGEO: International Society of Geographical and Epidemiologic Ophthalmology.

## Data Availability

Data described in the manuscript, codebook, and analytic code will not be made available because the data used in this study were from the NHANES database, which is a free and open database for all researchers around the world.

## Supplementary Materials

The following are available online at https://www.mdpi.com/article/10.3390/nu13124263/s1, Table S1: Summary of number and weight frequency for each diagnosis of glaucoma, Table S2: Comparison between the number of participants diagnosed by self-report and fundus image, Table S3: Comparison between the number of participants diagnosed by self-report and ISGEO, Table S4: Comparison between the number of participants diagnosed by ISGEO and fundus image.
