# Supplementary material for: Association between Daily Niacin Intake and Glaucoma: National Health and Nutrition Examination Survey
Source: Nutrients. 2021 Nov 26;13(12):4263. doi: 10.3390/nu13124263 (PMC8709149; doi:10.3390/nu13124263)
Supplement: Supplementary file 1 [file nutrients-13-04263-s001.zip › nutrients-1423443-supplementary.pdf]

Table S1. Summary of number and weight frequency for each diagnosis of glaucoma.

	Daily niacin intake	Glaucoma	Participants	Glaucoma Weight Frequency	Participants Weight Frequency
Self-reported	Quartile 1	122	1442	3,293,492.2	48,735,092.27
	Quartile 2	111	1442	4,230,737.3	58,179,306.59
	Quartile 3	83	1442	2,449,963.7	61,583,126.58
	Quartile 4	77	1442	2,886,335.8	72,293,042.40
	Total	393	5768	12,860,528.9	240,790,567.80
Fundus Image	Quartile 1	35	1036	1,118,830.5	36,299,097.51
	Quartile 2	34	1091	1,110,805.4	46,113,645.45
	Quartile 3	29	1188	710,460.4	54,012,436.69
	Quartile 4	27	1231	722,735.3	63,385,420.41
	Total	125	4546	3,662,831.7	199,810,600.10
ISGEO criteria	Quartile 1	29	1034	718,321.1	36,268,620.70
	Quartile 2	33	1089	1,075,492.9	46,015,148.46
	Quartile 3	26	1187	647,044.2	53,959,941.48
	Quartile 4	23	1229	755,337.3	63,358,489.31
	Total	111	4539	3,196,195.5	199,602,200.00

Table S2. Comparison between the number of participants diagnosed by self-report and fundus image.

		**Fundus image Criteria**			
		**No Glaucoma**	**Glaucoma**	**Not Eligible for Fundus Image Criteria**	**Total**
**Self-reported**	**No glaucoma**	4241	65	1069	5375
	**Glaucoma**	180	60	153	393
	**Total**	4421	125	1222	5768

Table S3. Comparison between the number of participants diagnosed by self-report and ISGEO.

		**ISGEO Criteria**			
		No glaucoma	Glaucoma	Not eligible for ISGEO criteria	Total
**Self-reported**	**No glaucoma**	4236	66	1073	5375
	**Glaucoma**	192	45	156	393
	**Total**	4428	111	1229	5768

Table S4. Comparison between the number of participants diagnosed by ISGEO and fundus image.

		Fundus Image Criteria			
		**No Glaucoma**	**Glaucoma**	**Not Eligible for Fundus Image Criteria**	**Total**
**ISGEO**	**No glaucoma**	4386	42	0	4428
	**Glaucoma**	32	79	0	111
	**Not eligible for ISGEO criteria**	3	4	1222	1229
	**Total**	4421	125	1222	5768
